# Prediction of Visual Acuity after anti-VEGF Therapy in Diabetic Macular Edema by Machine Learning

**DOI:** 10.1155/2022/5779210

**Published:** 2022-04-19

**Authors:** Ying Zhang, Fabao Xu, Zhenzhe Lin, Jiawei Wang, Chao Huang, Min Wei, Weibin Zhai, Jianqiao Li

**Affiliations:** ^1^Department of Ophthalmology, Qilu Hospital, Cheeloo College of Medicine, Shandong University, Jinan, China; ^2^Department of Occupational and Environmental Health, School of Public Health, Cheeloo College of Medicine, Shandong University, Jinan, China; ^3^Shandong Qidu Pharmaceutical Co. Ltd., Shandong Provincial Key Laboratory of Neuroprotective Drugs, Zibo, China; ^4^State Key Laboratory of Ophthalmology, Zhongshan Ophthalmic Center, Sun Yat-Sen University, Guangzhou, China

## Abstract

**Purpose:**

To predict visual acuity (VA) 1 month after anti-vascular endothelial growth factor (VEGF) therapy in patients with diabetic macular edema (DME) by using machine learning.

**Methods:**

This retrospective study included 281 eyes with DME receiving intravitreal anti-VEGF treatment from January 1, 2019, to April 1, 2021. Eighteen features from electronic medical records and measurements data from OCT images were extracted. The data obtained from January 1, 2019, to November 1, 2020, were used as the training set; the data obtained from November 1, 2020, to April 1, 2021, were used as the validation set. Six different machine learning algorithms were used to predict VA in patients after anti-VEGF therapy. After the initial detailed investigation, we designed an optimization model for convenient application. The VA predicted by machine learning was compared with the ground truth.

**Results:**

The ensemble algorithm (linear regression + random forest regressor) performed best in VA and VA variance predictions. In the validation set, the mean absolute errors (MAEs) of VA predictions were 0.137-0.153 logMAR (within 7-8 letters), and the mean square errors (MSEs) were 0.033-0.045 logMAR (within 2-3 letters) for the 1-month VA predictions, respectively. For the prediction of VA variance at 1 month, the MAEs were 0.164-0.169 logMAR (within 9 letters), and the MSEs were 0.056-0.059 logMAR (within 3 letters), respectively.

**Conclusions:**

Our machine learning models could accurately predict VA and VA variance in DME patients receiving anti-VEGF therapy 1 month after, which would be much valuable to guide precise individualized interventions and manage expectations in clinical practice.

## 1. Introduction

The International Diabetes Federation (IDF) has estimated that 536.6 million people worldwide suffered from diabetes mellitus (DM) while more than 140 million Chinese lived with DM in 2021 [1, 2]. Diabetic retinopathy (DR) remains the main complication of DM and a leading cause of blindness among working-age adults [1]. Diabetic macular edema (DME) is the most common cause of DR-related vision loss, which is characterized by the accumulation of fluid within the central retina and macular thickening caused by blood-retinal barrier dysfunction [3–6]. DME affects approximately 10% of patients with DR [6, 7] and leads to visual loss and reduction in quality of life, resulting in a substantial socioeconomic burden if left untreated [3, 7].

Laser photocoagulation is used to be recommended as the standard treatment protocol for DME, providing vision stabilization but with limited efficacy in improving vision [8]. Currently, anti-vascular endothelial growth factor (anti-VEGF) therapy has become the first-line treatment because anti-VEGF agents can reduce edema and thereby prevent further structural damage and improve vision [8, 9]. Two major anti-VEGF drugs commonly used and covered by the national basic medical insurance in China are ranibizumab and conbercept (Lumitin, Chengdu Kanghong Biotech Corporation Ltd., Chengdu, Sichuan, China) [10]. Many clinical studies indicated that anti-VEGF therapy was anatomically and functionally effective, with 30% of DME patients achieving significant improvement in best-corrected visual acuity (BCVA) [11]. However, not all patients respond satisfactorily to anti-VEGF injection; approximately 31.6-65.6% of patients do not respond or respond incompletely to anti-VEGF agents [12]. Switching to other potentially effective treatments at an early stage is recommended, such as intravitreal corticosteroid therapy for patients with a suboptimal response [13]. Thus, predicting the treatment response, differentiating patients who do not respond to anti-VEGF, and determining optimal treatment regimens are considered crucial.

Furthermore, DME as a chronic disease requires repeated injections of anti-VEGF drugs [14, 15]. Several anti-VEGF treatment regimens have been developed, such as monthly or bimonthly injections, pro re nata (PRN), and treat and extend (TAE) regimen [16]. PRN is the most common regimen in the treatment for DME to optimize the treatment effects and cost-effectiveness [17]. Many random clinical trials recommend three loading doses of anti-VEGF injections followed by a PRN regimen, namely, 3 + PRN [17]. But in the real world, three loading injections would be a great economic and psychological burden for patients with DME, especially in developing countries [18]. So, ophthalmologists often practice a 1 + PRN regimen, wherein patients received one anti-VEGF injection at the first month, and patients were observed monthly and received repeat injections based on BCVA and optical coherence tomography (OCT) imaging [18–21]. Thus, predicting the BCVA and OCT morphological features after anti-VEGF injection holds a huge value in the efficient management of DME.

OCT is the cornerstone of diagnosing, assessing, and managing DME [3]. Some OCT morphological features have been reported to be predictors for treatment outcome after anti-VEGF therapy for DME, including different OCT patterns of DME, the status of hyperreflective foci (HF), retinal inner layer, inner segment/outer segment (IS/OS), external limiting membrane (ELM), and central macular thickness (CMT) [22–24]. However, these parameters only estimated the likelihood of good or poor treatment response; an accurate prediction of BCVA after anti-VEGF therapy remains hard in clinical practice.

Machine learning (ML), which can rapidly manage a massive amount of information like images and clinical variables, is increasingly being used for the clinical prediction that is difficult for the physician in the field of ophthalmology [25–29]. ML-based algorithms can predict treatment response and treatment demand in diseases such as DR, DME, age-related macular degeneration (AMD), retinopathy of prematurity (ROP), and retinal vein occlusion (RVO) [30]. Liu et al. have been accurately predicted posttreatment BCVA at 1 month after three loading doses of anti-VEGF injections in DME patients using ML [16, 31]. However, it remains to be elucidated how it can predict posttreatment BCVA in 1 + PRN treatment regimens.

Thus, the purpose of this study was to predict VA at 1 month after anti-VEGF injection using ML based on clinical variables and OCT characters in DME patients with 1 + PRN treatment regimens. Furthermore, we optimized the prediction model by using fewer features to develop an optimization model suitable for clinical application.

## 2. Method

This retrospective observational study was approved by the Research Ethics Committee, Qilu Hospital, Shandong University. The protocol was performed following the Declaration of Helsinki. Informed consent was waived due to anonymous data extraction with no direct patient and public involvement in the study.

A total of 281 eyes were enrolled in the Department of Ophthalmology, Qilu Hospital, Shandong University from January 1, 2019, to April 1, 2021. The inclusion criteria were as follows: (1) DME diagnosed by OCT; (2) patients aged than18 years old; (3) patients received anti-VEGF therapy with 1 + PRN regimens (either ranibizumab 0.5 mg/injection or conbercept 0.5 mg/injection) during the study period; and (4) VA and OCT data were available pre- and postoperative (1 month). However, it is difficult to perform follow-up visits with a fixed date because the patients with DME are mostly working-age population with the different worksheets, so we determined a time range (1 month ±3 days) to ensure the accuracy of the study. Both eyes and each injection from the same patient were assessed independently. The eyes with any prior intravitreal pharmacotherapy, significant cataract, vitreous surgery, and other conditions that may influence visual acuity such as corneal opacity, glaucoma, macular degeneration, retinal vein occlusion, and other retinal pathologies were excluded from the study.

During data processing, clinical data such as gender, age, baseline VA, and 1-month VA after anti-VEGF injection, as well as the preoperative OCT image features were extracted manually. Quantitative evaluations of OCT images were evaluated on horizontal OCT images within a 3000 *μ*m diameter around the foveal center, including central macular thickness (CMT), number of cysts, and the shortest distance of cysts from the fovea, the vertical length of the largest cyst. The CMT was defined as the distance between the internal limiting membrane and the retinal pigment epithelium (RPE) at the fovea. The number of intraretinal cystoid spaces with a width larger than 50 *μ*m was counted. All the features were measured using the caliper tool built into the OCT software (Zeiss) by two experienced technicians (Y Zhang and M Wei) who were masked to any clinical data of the patients; no significant difference was found between the different datasets by calculating the Pearson correlation. The following morphologic features were also assessed, including diffuse retinal thickening (DRT), cystoids macular edema (CME), serous retinal detachment (SRD), posterior hyaloid traction (PHT), traction retinal detachment (TRD), disorganization of retinal inner layer (DRIL), disruption of external limiting membrane (ELM), disruption of inner segment/outer segment (IS/OS), the presence of epiretinal membrane (ERM), exudation or hemorrhage, and hyperreflective foci (HF).

This batch of data consists of 281 samples, which are composed of 18 features and 2 labels. These features are almost nonlinear data. For the VA prediction, all baseline data were analyzed and processed, and the proportion of the training set and validation set were divided into 80% and 20%, according to the admission time, respectively.

**Figure 1:**
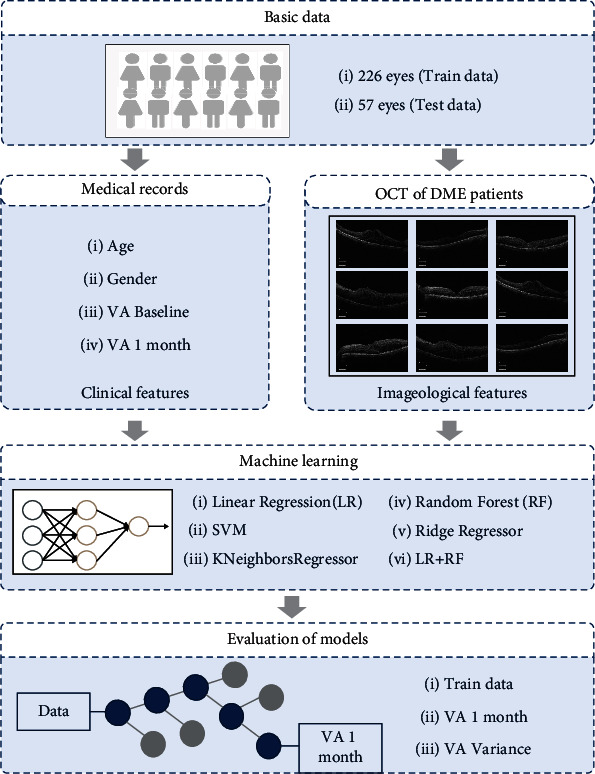
Overall study workflow. Workflow diagram showed the training overview for the visual acuity prediction model.

To predict the logMAR VA of patients with anti-VEGF therapy, we tested six regression algorithms with state-of-the-art performance in each adaptive domain. They are listed as follows: linear regression (LR), SVM, K neighbors regressor, random forest regressor (RF), ridge regressor, and LR + RF [26–28, 32–34]. And we established and optimized the LR + RF with the data. The workflow diagram is shown in Figure 1.

To quantitatively evaluate the model performance, we applied two evaluation indicators, mean absolute error (MAE), and mean square error (MSE). MAE is calculated as the average value of the absolute error of the prediction results, which directly reflects the deviation of the predicted values from the actual values and shows the capability of the models. The MSE is calculated as the average value of the square of the error of the prediction results, which showed the robustness of the models.

**Figure 2:**
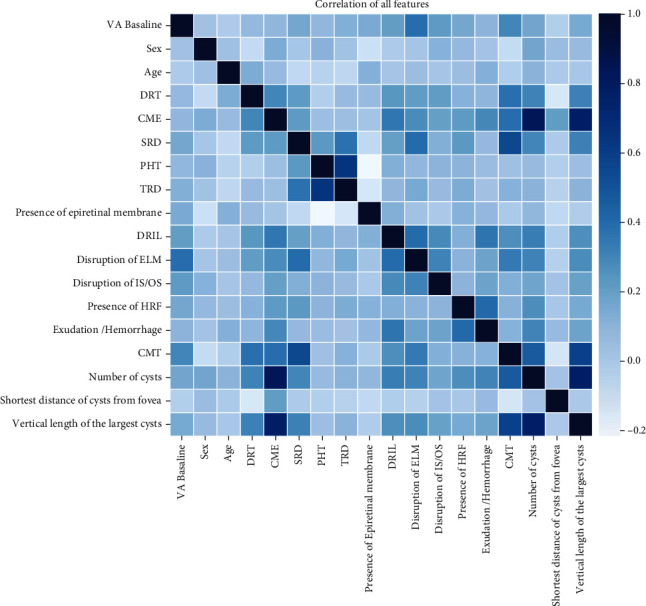
Correlation matrix of all baseline data. A correlation matrix was established for all baseline data to exclude redundant features.

**Figure 3:**
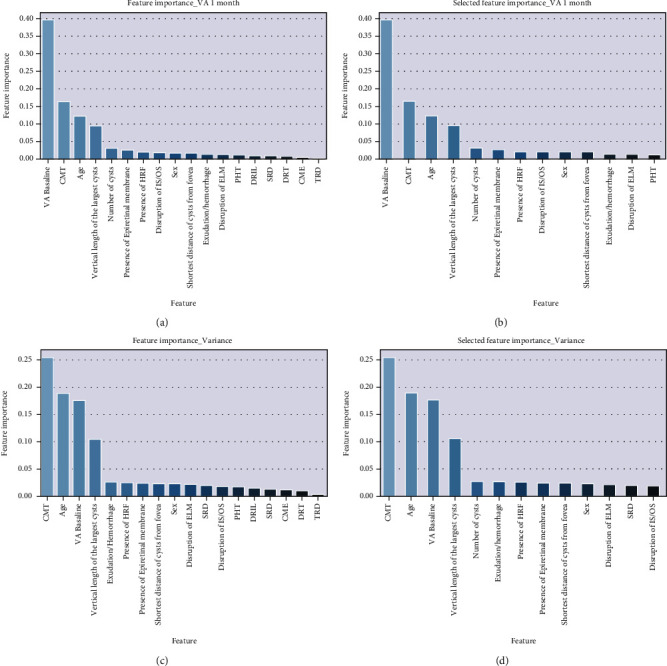
Relative importance of different features for VA and VA variance predictions. The plot showed the weight of different features for the VA (a, b) and VA variance (c, d) prediction task. The blue bar indicated how important the feature was for the model on the different test runs.

At the beginning of the 1-month VA prediction, a correlation matrix was established for all baseline data to ensure that there were no redundant features (Figure 2). We found that the CME was significantly correlated with the number of cysts and the vertical length of the largest cyst during the data processing. Then, the random forest algorithm was established to output the feature importance, which showed that CME and the proportion of the other 4 features' importance were less than 0.01 (Figure 3). After the initial data exploration, 5 features were deleted, and 13 features were retained for model training.

During the training process, a grid-search module was applied to optimize the model parameters. The experimental results showed that the LR and RF models trained by the selected features performed best. To evaluate the performance of these two models, the VA values predicted by the LR and RF were compared with the values predicted by the other algorithms, including SVM, K neighbors regressor, and ridge regressor. In addition, these two models were further combined into an ensemble algorithm by applying a stacking integration framework to obtain a model with better predictive performance and robustness. At the first layer of stacking, a random forest regression model is trained by the selected data. And then, the first layer outputs and the training set were then put into the linear regression model for training; thus, a combined LR + RF model based on stacking framework is obtained.

After completing the initial detailed investigation, we designed another model that would be more conveniently applied for VA variance predictions. The variance of VA was defined as the difference value between 1-month visual acuity and baseline visual acuity. The steps of data processing, the algorithms used, and the model evaluation procedure were the same as in the previous model.

## 3. Result

Descriptive data of the study population are provided in Table 1. The VA values (logMAR) predicted by machine learning models were compared to the ground truth (i.e., the outcome as recorded during follow-up) (Tables 2). In the comparative analysis, the ensemble algorithm (linear regression + random forest regressor) exhibited the highest accuracy in VA and VA variance predictions, outperforming the linear regression, SVM, K neighbors regressor, random forest regressor, and ridge regressor models. So, all subsequent analyses were conducted based on the ensemble algorithm.

**Figure 4:**
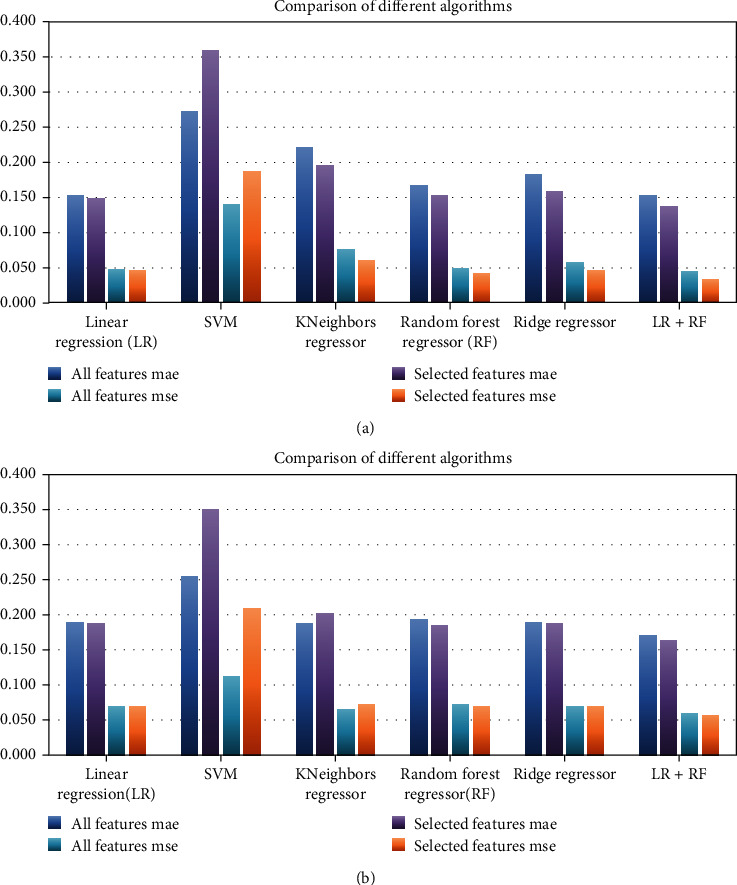
Comparison of VA and VA variance predictions in different models. The plot showed the performance of the different algorithms for the VA (a) and VA variance (b) prediction task.

The predicted values were compared with the closest VA measurements available. In the first model, when all data collected from electronic medical records and OCT were considered, the MAEs of the VA predictions with respect to the ground truth were 0.153logMAR (within8 letters), and the MSEs of the VA predictions with respect to the ground truth were 0.045logMAR (within 3 letters), respectively. The optimization model achieved a comparable level of predictive power with fewer features, the MAEs of the VA predictions with respect to the ground truth were 0.137 logMAR (within 7 letters), and the MSEs of the VA predictions with respect to the ground truth were 0.033 logMAR (within 2 letters), respectively. The details of predictions with SVM, K neighbors regressor, random forest regressor, and ridge regressor models are shown in Table 2. Besides, to predict the response of vision more detailed after anti-VEGF, we built another model to predict the changes of VA after treatment; accuracy of VA variance prediction at 1 month is shown in Table 3 and Figure 4.

**Figure 5:**
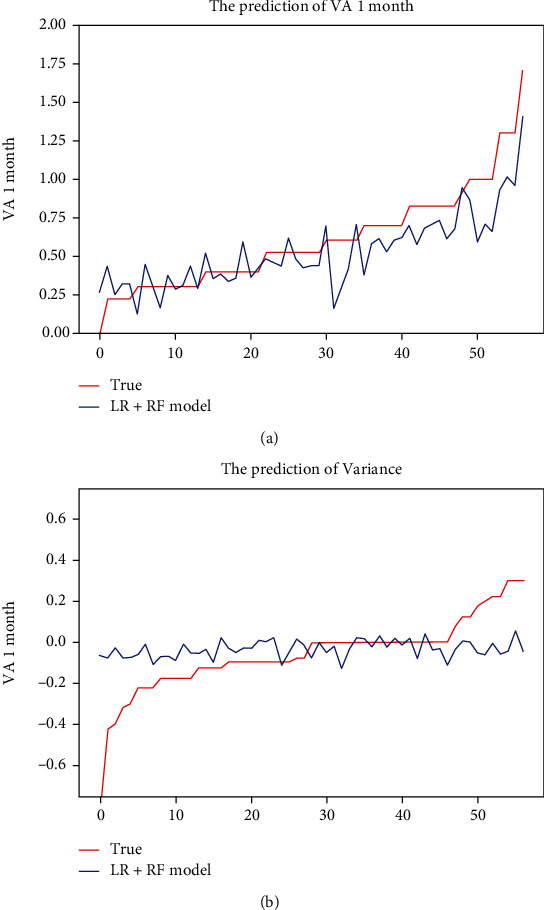
Visual acuity prediction fitting curve in different models. (a) fitting curve for VA predictions; (b) fitting curve for VA variance predictions.

Figure 5 showed the differences between the predicted and ground truth VA values, which were the VA values measured 1 month after anti-VEGF. The VA measured at baseline was the most important predicted factor for 1-month VA prediction in both original and optimization models. However, in the prediction of the variance of VA, the CMT and age were the most important predicted factors. Details of feature importance are shown in Figure 3.

## 4. Discussion

The present study developed an ensemble ML system to predict VA at 1 month after anti-VEGF injection in DME patients treated with 1 + PRN regimens and an ensemble model for the variance of VA prediction. We demonstrated that the VA can be accurately predicted within a very small error of 2-8 letters (1-2 lines on EDTRS visual chart) based on18 features from electronic medical records and OCT images using ML algorithms. Based on these two models, we could accurately predict the visual benefit of DME patients treated with 1 + PRN regimens, evaluate the efficacy of the anti-VEGF therapy, and determine whether to change the treatment strategy.

With the increasing number of DME patients and anti-VEGF injections worldwide, it is crucial to make cost-effective treatment regimens to prevent visual loss and reduce the economic and time burden [16, 17]. The 1 + PNR regimen, which used monthly evaluation either clinical or imaging and as-needed treatment to reduce the number of injections, demonstrated a noninferior visual improvement at 1 year along with significantly fewer injections [18–20]. Real-world data show a poor compliance with treatment among DME patients, and patients undergo lower treatment intensity of anti-VEGF injection compared with patients in random clinical trials [14, 15]. This is partly because patients are uncertain of treatment outcomes and anxious about the high cost of anti-VEGF therapy [14, 15]. So, an accurate prediction of posttreatment VA for anti-VEGF therapy is paramount to obtaining better compliance for multiple anti-VEGF injections, especially in 1 + PRN regimens. It can not only help ophthalmologists to make better treatment plans but also help patients to reduce psychological pressure and manage the expectations.

Previous studies have investigated the predictive factors of treatment outcomes after anti-VEGF therapy in eyes with DME, including OCT data and clinical data [18, 31, 35–38]. OCT images are essential for managing the treatment strategy of DME, which provides a way to objectively detect the treatment response [22–24, 35]. Data obtained from OCT images, such as different OCT morphologic characteristics of DME, retinal thickness, the height of cysts, DRIL, hyperreflective foci, and the integrity of ELM and IS/OS, have been shown as the predictors of treatment responses following anti-VEGF treatment [22–24, 35]. In the present study, our results showed that CMT, vertical height of the largest cysts, and the number of cysts were important predictive factors for VA prognosis in patients with DME. It was an advantage that CMT and the intraretinal cysts on OCT images were easily evaluated visually during clinical practice.

In the real world, exhaustive clinical data collection is not cost-effective and does not adapt properly to clinical practice. Although several baseline demographics and ocular findings were evaluated, the treatment benefit was found to associate with only a few features, such as age, gender, baseline VA, HbA1c levels, and prior panretinal photocoagulation (PRP) [36, 37]. However, some studies showed that there was a lack of correlation between HbA1c and posttreatment VA because real-time HbA1c levels reflected the blood glucose control in the previous two months and not prospectively after administering treatment [38]. Furthermore, it is unknown why prior PRP is negatively associated with improvement of VA, which may be due to the macular ischemia [38]. Since PRP is a part of the treatment regimen, it is not possible to separate the effect of PRP from anti-VEGF treatment on the VA outcomes [8]. Thus, only three clinical variables including age, gender, and baseline VA were used to develop the predictive model in this study. Baseline VA and age were found to have a critical impact on visual outcomes following anti-VEGF treatment in DME.

In addition to appropriate predictors, the applications of algorithms were equally important for VA prediction. Linear regression is the process of estimating unknown values based on multiple known data [27, 39]. It assigns a factor weight to each known data so that the final prediction results approximate the real results [27]. In the fitting, the most appropriate weight parameters are assigned to each feature to achieve prediction results more accurately. Random forest is an ensemble learning algorithm, which is emerging among the other algorithms mainly resulting in its random and forest structure [27, 33]. On the one hand, its bootstrap mechanism can resist overfitting since the sample training is stochastic. On the other hand, forest is a combination of tree structures that could fit nonlinear data more accurately [33]. With the statistics of feature importance in random forest, it shows that some features are more advantageous for the prediction [27, 33]. The experiment result “MAE and MSE” shows that the screened features on all models compared with all data improved significantly, especially linear regression and random forest.

What's more, due to the advantages of linear regression and random forest, the stack architecture was used to combine the two models [25, 32, 33]. Firstly, the random forest could be utilized to extract the hidden information of some important features, and then these data can be fitted with the final output prediction results by linear regression [32, 33]. LR + RF model has better indicators in comparing among any single model, and it can be seen that the prediction of the model is relatively close to the true value in the fitting curve.

There are several limitations in our study. The first limitation is that the sample size was rather small and more and longer follow-up data are still necessary to improve the performance of our prediction models. We could try to predict the response of anti-VEGF in patients with DME using deep learning with more paired OCT images. Secondly, extracting measurement data manually from OCT is a very labor-intensive and time-consuming undertaking. With the additional OCT data, we will likely be able to use more data-hungry approaches like deep learning for predicting VA.

An accurate prediction of posttreatment visual response to anti-VEGF therapy remains challenging in clinical practice. ML could accurately predict VA and VA variance 1 month after the anti-VEGF therapy in DME patients treated with 1 + PRN regimens, and the optimization model could be more easily applied by ophthalmologists. This will be invaluable to guide precise individualized interventions and manage expectations.

## Figures and Tables

**Table 1:** Patient demographics.

Demographics	Training data	Test data	*P* value
Eyes	226	55	N/A
Age (years)	56.57 ± 10.12	53.38 ± 10.41	0.89
VA (baseline)	0.585 ± 0.316	0.576 ± 0.332	0.90
VA (endpoint)	0.540 ± 0.342	0.536 ± 0.331	0.94
VA (variance)	−0.029 ± 0.265	−0.031 ± 0.271	0.90
Diffuse retinal thickening	153	38	N/A
Cystoids macular edema	114	29	N/A
Serous retinal detachment	56	16	N/A
CMT	358.36 ± 225.39	348.42 ± 221.47	0.88
DRILL	195	41	N/A
Presence of HRF	53	15	N/A
Exudation or hemorrhage	112	29	N/A

VA, visual acuity; values are presented as the means ± standard deviations at baseline in different groups (in logarithm of minimum angle of resolution [logMAR] units). CMT, central macular thickness; DRIL, disorganization of retinal inner layer; HRF, hyperreflective foci.

**Table 2:** Accuracy of visual acuity predictions.

Algorithm learner	MAE		MSE	
	All features	Selected features	All features	Selected features
Linear regression (LR)	0.153	0.149	0.048	0.046
SVM	0.272	0.359	0.140	0.188
K neighbors regressor	0.222	0.195	0.076	0.060
Random forest regressor (RF)	0.168	0.153	0.050	0.042
Ridge regressor	0.183	0.159	0.058	0.046
LR + RF	0.153	0.137	0.045	0.033

MAE, mean absolute error; MSE, mean square error; accuracy (VA in logMAR) of VA prediction at 1 month after anti-VEGF compared with the ground truth.

**Table 3:** Accuracy of visual acuity variance predictions.

Algorithm learner	MAE		MSE	
	All features	Selected features	All features	Selected features
Linear regression (LR)	0.188	0.188	0.069	0.069
SVM	0.254	0.349	0.111	0.208
K neighbors regressor	0.187	0.202	0.065	0.071
Random forest regressor (RF)	0.193	0.185	0.071	0.069
Ridge regressor	0.188	0.187	0.069	0.069
LR + RF	0.169	0.164	0.059	0.056

MAE, mean absolute error; MSE, mean square error; accuracy (VA in logMAR) of VA variance prediction at 1 month after anti-VEGF compared with the ground truth.

## Data Availability

The data used to support the findings of this study are available from the corresponding author upon reasonable request.
